# Lumpy skin disease: Attempted propagation in tick cell lines and presence of viral DNA in field ticks collected from naturally-infected cattle

**DOI:** 10.1016/j.ttbdis.2014.11.002

**Published:** 2015-03

**Authors:** E.S.M. Tuppurainen, E.H. Venter, J.A.W. Coetzer, L. Bell-Sakyi

**Affiliations:** a The Pirbright Institute, Ash Road, Pirbright, Surrey GU24 0NF, United Kingdom; b Department of Veterinary Tropical Diseases, Faculty of Veterinary Science, University of Pretoria, Private Bag X04, Onderstepoort 0110, South Africa

**Keywords:** Lumpy skin disease virus, Ticks, Tick cell lines, *Rhipicephalus*

## Abstract

Lumpy skin disease (LSD) is of substantial economic importance for the cattle industry in Africa and the Near and Middle East. Several insect species are thought to transmit the disease mechanically. Recent transmission studies have demonstrated the first evidence for a role of hard (ixodid) ticks as vectors of lumpy skin disease virus (LSDV). The aim of this study was to attempt *in vitro* growth of the virus in *Rhipicephalus* spp. tick cell lines and investigate *in vivo* the presence of the virus in ticks collected from cattle during LSD outbreaks in Egypt and South Africa. No evidence was obtained for replication of LSDV in tick cell lines although the virus was remarkably stable, remaining viable for 35 days at 28 °C in tick cell cultures, in growth medium used for tick cells and in phosphate buffered saline. Viral DNA was detected in two-thirds of the 56 field ticks, making this the first report of the presence of potentially virulent LSDV in ticks collected from naturally infected animals.

## Introduction

Lumpy skin disease (LSD) is an economically important generalised skin disease of cattle, caused by the lumpy skin disease virus (LSDV) of the genus *Capripoxvirus* (Buller et al., 2005). The disease was originally reported from sub-Saharan Africa in 1929 and it is currently endemic across Africa and in most parts of the Middle East. The most recent outbreaks were reported in late 2013 from Turkey and Iraq. In May 2014 the disease was reported for the first time in Iran and in July 2014 in Azerbaijan (OIE WAHID database). The main mode of transmission of LSDV is *via* arthropod vectors whereas direct or indirect contact between infected and susceptible animals is an inefficient method of transmission (Carn and Kitching, 1995; Weiss, 1968). In order to effectively control the spread of the disease, it is necessary to fully understand the role of different arthropod species in LSDV transmission. Previous studies have demonstrated the ability of *Aedes aegypti* mosquitoes to transmit the virus (Chihota et al., 2001). Stable flies (*Stomoxys calcitrans*) have been shown to be competent vectors for sheeppox virus (Kitching and Mellor, 1986), another capripoxvirus. Although many other insect species are likely to be mechanical vectors of LSDV, no other clinical transmission trials on possible insect vectors of LSDV have been carried out.

The vector capacity of hard ticks has recently been under intense investigation. Mechanical transmission of LSDV by male *Rhipicephalus appendiculatus* ticks was demonstrated (Tuppurainen et al., 2013b). After feeding on experimentally-infected cattle, semi-engorged male ticks were transferred onto naïve animals, which became viraemic and seroconverted. In another study, evidence of mechanical transmission was obtained for *Amblyomma hebraeum* males, although no seroconversion was detected and the level of viraemia was very low (Lubinga et al., 2013a). Presence of viral nucleic acid was demonstrated at the feeding sites of *A. hebraeum* and *R. appendiculatus* males in the recipient animals (Tuppurainen et al., 2011). Due to their large mouthparts and interrupted feeding pattern, it is most likely that *Amblyomma* males are equally important as mechanical vectors of LSDV as *Rhipicephalus* males.

Using PCR and virus isolation, the presence of the virus has been detected in experimentally-induced saliva samples collected from *A. hebraeum* and *R. appendiculatus* males after feeding on LSDV-infected cattle. In the same study it was demonstrated that in both tick species the virus also survived the process of moulting to adults, following feeding of nymphs on LSDV-infected cattle. Before the salivation was induced, newly-moulted adults were allowed to harden for approximately one month and saliva samples then tested positive using real-time PCR and virus isolation (Lubinga et al., 2013b).

Lumpy skin disease virus has been detected in eggs (Tuppurainen et al., 2011) and larvae (Lubinga et al., 2014c) originating from *Rhipicephalus (Boophilus) decoloratus* females fed on LSDV-infected hosts. When these larvae were allowed to feed on two naïve cattle they became viraemic and developed skin lesions which tested positive for LSDV using real-time PCR (Tuppurainen et al., 2013a). Larvae hatched from eggs laid by *A. hebraeum* females previously fed on infected cattle also tested positive by real-time PCR and virus isolation (Lubinga et al., 2014c), although the recipient animal used for feeding these larvae did not show clinical signs or seroconversion. Evidence of vertical transmission of the virus by *R. appendiculatus* has been published (Lubinga et al., 2014c). In transstadially and intrastadially-infected adult *R. appendiculatus* and *A. hebraeum* ticks the presence of the virus was also demonstrated using immunohistochemical staining in various tick organs including midgut, salivary glands, ovaries, testes, and fat body, demonstrating that the virus was able to pass from the midgut into the haemocoel. Interestingly the presence of the virus was demonstrated in tick tissues that do not undergo histolysis such as synganglia and haemocytes, and in tissues which develop during the moulting process such as reproductive organs (Lubinga et al., 2014a).

In studies investigating potential biological transmission by ticks, the results were based mainly on PCR findings and to a lesser extent on virus isolation. When isolation of virus was carried out from tick samples, the presence of LSDV indicated by suspected cytopathic effect (CPE) was confirmed by testing infected mammalian cell cultures by real-time PCR. The resultant cycle threshold (C_t_) values varied between 35 and 39 indicating the presence of viral DNA but not necessarily active replicating virus. Therefore, an important question still remains: does the virus replicate in tick cells? The present study was carried out to investigate replication and/or survival of LSDV *in vitro* in cell lines derived from the tick species *R. appendiculatus*, *Rhipicephalus evertsi* and *R. (B.) decoloratus*. In addition, ticks of several species were collected from naturally-infected cattle during LSDV outbreaks in Egypt and South Africa and tested for the presence of LSDV using PCR and virus isolation.

## Materials and methods

### Virus isolate

The LSDV used in this study was isolated from a skin nodule collected from a bull experimentally infected with a South African LSDV isolate (SA 248/93). The virus was passaged twice on bovine dermis primary LB9.D cells (LGC Promochem, Teddington, UK). The titre of the virus was 6.5 log TCID_50_/ml. The volume of virus inoculum for each tick cell culture tube was 200 μl and the day of inoculation is referred to as Day 0 post-infection (pi).

### Tick cell lines and growth medium

The tick cell lines used in this study, the origin of the cells, growth medium used for each cell line and references are listed in Table 1. All cell lines were propagated in 2 ml growth medium in ambient air in sealed flat-sided culture tubes (Nunc) at 28 °C with weekly medium changes (removal and replacement of 1.5 ml medium). The embryo-derived cell lines RAE/CTVM1 *(R. appendiculatus)*, REE/CTVM29 *(R. evertsi)* and BDE/CTVM16 *(R. (B.) decoloratus)* and the developing adult-derived cell line RA243 (*R. appendiculatus*) were grown in L-15 (Leibovitz) medium (PAA Laboratories, Yeovil, UK) supplemented with 10% tryptose phosphate broth, 20% foetal bovine serum (FBS), 2 mM l-glutamine (l-glut), 100 units/ml penicillin and 100 μg/ml streptomycin (L-15). The embryo-derived cell line BDE/CTVM14 *(R. (B.) decoloratus)* and the developing adult-derived cell line RAN/CTVM3 (*R. appendiculatus*) were grown in Hanks’ balanced salt solution (Sigma–Aldrich, Gillingham, UK) supplemented with 0.5% lactalbumin hydrolysate (Sigma–Aldrich, Gillingham, UK), 20% FBS, l-glut and antibiotics as above (H-Lac). Subcultures were carried out when required by adding fresh growth medium, re-suspending the cells and dispensing 2 ml volumes into fresh culture tubes while leaving 2 ml cell suspension in the parent tube.

### Infection of tick cell cultures with LSDV

Inoculation of LSDV onto tick cell lines was carried out in triplicate. Four tubes of each cell line were seeded on Day-4. On Day 0, 200 μl of virus suspension was added directly to the growth medium of three of the tubes containing tick cells, and to cell-free control tubes containing only either growth medium (L-15 or H-Lac) or phosphate buffered saline (PBS). The fourth tube of each cell line was used as an uninfected negative control culture.

The optimal growth temperature for the tick cell lines used in this study is 28 °C which is likely to be a lower than optimum temperature for the virus to replicate. In the first experiment, in an attempt to increase the adaptation of the virus to replication in tick cells, infected and uninfected tick cells as well as LSDV-infected L-15 control tubes were incubated first for seven days at 37 °C followed by four weeks at 28 °C. Because it was known that the tick cells would not survive at 37 °C for long (author's unpublished observation), another group was incubated at 28 °C for 35 days.

In the second experiment, infected and uninfected tick cells and infected L-15 (C1), H-Lac (C2) and PBS (C3) control tubes were incubated at 28 °C for 35 days. Additional control tubes C1D and C2D were identical to C1 and C2 except medium changes were carried out at the same time and in the same manner as those of the tubes containing tick cells.

Medium was changed weekly on Days 6, 13, 20, 27 and 34 pi in all tick cell culture tubes, maintaining a constant volume of 2 ml throughout the experiment: the tubes were held upright for a few minutes to allow floating cells to sink to the bottom, 1.5 ml spent medium was removed without disturbing the cell pellet, 1.5 ml fresh growth medium was added and the tubes were incubated horizontally.

All tick cell cultures were monitored by weekly inverted microscope examination; general appearance and density of LSDV-infected cultures was compared with that of uninfected control cultures.

### Sample collection

In order to determine the baseline C_t_ values and virus titres, samples were collected from all inoculated tubes immediately after adding the virus inoculum on Day 0 pi and then on Day 35 pi. Prior to sample collection, cells attached to the cell culture tube were gently detached by flushing with the growth medium, the cell suspension was mixed well and a 200 μl sample was collected. In order to disrupt the cells, samples were then sonicated using a Thistle Scientific Branson Sonifier 150 at a power setting of 2. Cell debris was spun down by centrifugation at 600 × *g* for 5 min at room temperature and the supernatants were collected and stored at −80 °C until tested.

### Collection of ticks from naturally-infected cattle during LSDV outbreaks

In September 2006, four semi-engorged *Rhipicephalus (Boophilus)* sp. female ticks were collected from three Holstein-Friesian cattle, recovering from LSD and still showing some skin lesions and scabs, at two privately owned dairy farms in Menofilia Governorate in Egypt. The collected ticks were transported to The Pirbright Institute in PBS with 10% glycerol at room temperature and stored at −20 °C until tested in November 2006. In March 2007, adult *Rhipicephalus*, *Amblyomma* and *Hyalomma* spp. ticks were collected from infected *Bos indicus* cross cattle from three smallholdings near Pretoria, Gauteng Province, South Africa and from cattle brought to a dip tank station in the same area. Between March and May 2013, *Amblyomma* and *Rhipicephalus (Boophilus)* ticks were collected from Sanga cattle at several dip tank stations in the Mnisi community area which lies in the north-eastern corner of the Bushbuckridge Municipal Area, Mpumalanga Province, in close proximity to the Kruger National Park border in South Africa. All ticks collected from South African cattle were placed in cryo tubes without any medium and transported in dry ice. The tick samples were stored at −80 °C without medium. Samples collected in 2007 were tested in early 2012. The tick samples originating from the LSDV outbreak in 2013 were tested nine months later. Ticks were identified where possible to species level. For ticks collected from South Africa in 2007, the gender and degree of engorgement of the ticks were recorded.

### Preparation of field ticks for real-time PCR and virus isolation

Prior to testing, ticks were washed with 70% ethanol and then rinsed with Schneider's Drosophila Medium (Lonza, Walkersville, MD, USA) containing 200 IU/ml penicillin, 200 μg/ml streptomycin and 0.5 μg/ml amphotericin B. Ticks were then cut into small pieces and snap-frozen in liquid nitrogen for 5 min before the samples were mixed with 500 μl of Dulbecco's Modified Eagle's medium (DMEM) The samples were then lysed using a Qiagen Tissue Lyser with 3 mm Tungsten beads at 25 Hz for 2 × 30 s. A further 500 μl of DMEM was added to the samples, followed by centrifugation at 600 × *g* for 5 min at room temperature and then supernatant was collected. One half of the supernatant was used for PCR; DNA was extracted using a previously-described DNA extraction method (Tuppurainen et al., 2011) followed by conventional PCR (Tuppurainen et al., 2011) or the real-time PCR (Bowden et al., 2008; Stubbs et al., 2012) as described below for testing the cell culture samples. In the real-time PCR, cycle threshold (C_t_) values over 39 were considered negative. The other half of the supernatant of each sample was used for virus isolation (Lubinga et al., 2014c). Tick samples collected from Egypt in 2006 did not contain sufficient material for virus isolation.

### DNA extraction and real-time PCR method for tick cell culture samples

In order to standardise the DNA extraction method, 50 μl of each cell culture sample was extracted using a robotic extraction technique (BioRobot Universal System, Qiagen). The presence of viral DNA in the samples was quantified using a previously-described general capripoxvirus real-time PCR (Bowden et al., 2008; Stubbs et al., 2012). Primers and a probe were used in combination with a QuantiFast Probe PCR Kit (Qiagen, Crawley, UK) in a Mx3005p Multiplex Quantitative PCR System (Strategene, Netherlands) (Bowden et al., 2008; Stubbs et al., 2012). This real-time PCR assay targets an 89 bp region within the LSDV P32 gene and utilises forward primer 5′-AAA ACG GTA TAT GGA ATA GAG TTG GAA-3′, reverse primer 5′-AAA TGA AAC CAA TGG ATG GGA TA-3′ and TaqMan probe 5′-6FAM-TGG CTC ATA GAT TTC CT-MGB/NFQ-3′. The assay is validated for use as a primary diagnostic method for the detection of LSDV DNA from samples submitted to the OIE Capripoxvirus Reference Laboratory at The Pirbright Institute, and is an ISO 17025 accredited method. A cut-off limit of C_t_ 39 (Stubbs et al., 2012) was used.

### Virus titration

The tick cell culture samples and cell-free control samples were titrated in 96-well flat bottom microtiter plates on bovine dermis cells LB9.D (LGC Promochem, Teddington, UK) at 0.5 log dilutions from 10^−1^ to 10^−6^. Infected wells were identified by microscopic detection of CPE in cell monolayers. TCID_50_/ml values were calculated according to the Spearman–Kärber method (Kärber, 1931; Spearman, 1908).

## Results

In the first experiment, incubation of infected tick cells at the higher temperature of 37 °C for the first seven days did not have any effect on the amount of virus detected in the cultures four weeks later (Table 2). For RAE/CTVM1 and REE/CTVM29 cells, whether grown at 37 °C/28 °C or at constant 28 °C, the final C_t_ values were practically identical (average C_t_ 27.5) and in all cases higher than the Day 0 baseline values, indicating loss of virus from the cultures during the five-week period. However, in both *R. (B) decoloratus* groups the virus survived poorly in comparison with survival in *R. appendiculatus* and *R. evertsi* cells (Table 2). The titre of the original virus inoculum (SA 248/93) was 6.5 log TCID_50_/ml. The average Day 0 baseline titre in tick cell culture tubes was 5.6 log TCID_50_/ml, consistent with the 1 in 10 dilution resulting from inoculation into the cultures. After 35 days the titre of the virus in RAE/CTVM1 kept at 28 °C for 35 days was 3.34 log TCID50/ml whereas in REE/CTVM29 and BDE/CTVM16 cells the titre was below the detection limits of the test.

There was no change in the C_t_ values between Days 0 and 35 pi of virus incubated in L-15 growth medium. Incubation at 37 °C/28 °C resulted in a small increase in C_t_ (from 22.6 to 24.1) over 35 days but this increase was considerably lower than those seen in the infected tick cell cultures (Table 2).

As incubation at the higher temperature at the beginning of the first experiment did not have any detectable effect on the virus, a second experiment was carried out with incubation at 28 °C throughout. Three *R. appendiculatus* and two *R. (B.) decoloratus* cell lines, with cell-free medium and PBS controls, were infected with LSDV as described above and tested on Days 0 and 35 pi. The baseline and final C_t_ values and virus titres of the infected cell lines and controls are presented in Table 3. The baseline C_t_ values (Day 0 pi) for all five cell lines fell between 23.4 and 25.5. After 35 days, the C_t_ values for the three *R. appendiculatus* cell lines and the *R. (B.) decoloratus* cell line BDE/CTVM14 had risen to between 30.1 and 30.8, while the mean C_t_ value obtained for the *R. (B.) decoloratus* line BDE/CTVM16 was considerably higher at 34.09. At the end of the experiment for both *R. (B.) decoloratus* cell lines the virus titres were below the detection limits of the assay, although in BDE/CTVM16 cells live virus was growing in one out of the six wells of the 10^−1^ dilution. Interestingly, the *R. appendiculatus* cell lines grown in L-15 medium (RAE/CTVM1 and RA243) seemed to support the viability of the virus better than the cell line grown in H-Lac medium (RAN/CTVM3). This was reflected in the approximately 1 log^10^ lower virus titres in the H-Lac medium controls compared to the L-15 medium controls (Table 3). The C_t_ values of all the undiluted controls – C1 (L-15), C2 (H-Lac) and C3 (PBS) – showed little or no increase between Days 0 and 35 pi, indicating that viral DNA remained intact. However virus viability decreased in all three undiluted controls by between 0.76 and 2.33 log TCID_50_/ml and by an additional 0.83–1.34 log TCID_50_/ml in the two diluted medium controls C1D and C2D, demonstrating that detection of DNA by PCR does not necessarily reflect the viability of the virus.

### Presence of lumpy skin disease virus in ticks collected from naturally-infected cattle

The PCR results of LSDV in field ticks are presented in Table 4.

*Egypt*: The four semi- or fully engorged female ticks were identified to genus level as *Rhipicephalus (Boophilus)*. All four tested positive for LSDV using the conventional, gel-based PCR method, but there was not sufficient sample material for virus isolation.

*Pretoria, Gauteng, South Africa*: Out of nine *R. appendiculatus* females collected from cattle with severe manifestations of LSD, six were flat and three semi-engorged. All of them tested positive in real-time PCR (average C_t_ 35.8). Two partially-fed *R. appendiculatus* males collected from the same animals gave a slightly lower average C_t_ of 33.3. Attempts to isolate live virus from these samples were unsuccessful.

A total of 11 *R. (B.) microplus* females were collected for testing, two of which were feeding on animals severely infected with LSDV and nine of which were collected from cattle at the dip tank station. Some of the latter cattle were reported to be vaccinated against LSDV although no vaccination records were presented. None of the animals at the dip station had multiple skin lesions. One of the two *R. (B.) microplus* females from severely infected animals and three of the nine other ticks tested positive, with an average C_t_ value of 35.5.

Six male and three female *A. hebraeum* ticks were collected from one calf with scabs left from previous skin lesions. All the males tested positive (mean C_t_ 35.3). Only one out of three females, tested positive (C_t_ 37.3). Two semi-engorged *Hyalomma truncatum* females were also collected from this animal. Another two were collected from cattle at the dip tank station. The mean C_t_ value of 33 was obtained from *Hyalomma* females.

*Mnisi, Mpumalanga, South Africa*: A total of six *Amblyomma* ticks were collected from cattle at several dip tank stations and two of them tested positive (mean C_t_ 33.6). From a total of 11 *R. (Boophilus)* females, six ticks tested positive (mean C_t_ 32). Live virus was not isolated from any of the tick samples.

## Discussion

The aim of this study was to determine if LSDV is able to replicate in *Rhipicephalus* spp. tick cell lines and to investigate the presence of LSDV in ticks collected from infected cattle in the field. Although titration in mammalian cells did not indicate any increase in viral titre that would correspond to virus replication, viable infective LSDV survived for 35 days, albeit with some loss of titre, in three *R. appendiculatus* cell lines, tick cell growth medium and in PBS. Viral DNA survived at similar levels in a *R. evertsi* cell line. In contrast, much lower levels of viral DNA was obtained in *R. (B.) decoloratus* cell lines but the presence of extremely low-titre infective LSDV in one of the two cell lines was detected by virus titration after 35 days *in vitro*.

All capripoxviruses are known to grow slowly in mammalian cell culture and sometimes two to three passages at intervals of 10–12 days are required to successfully propagate these viruses (Weiss, 1968), particularly when they are isolated from samples containing a low number of viral particles. Usually CPE caused by LSDV infection cannot be detected visually before Day 4 pi and in some cases the appearance of CPE may take up to 14 days (Tuppurainen et al., 2005). Due to the physiological differences between tick and mammalian cells *in vivo*, LSDV replicating in skin or blood cells of cattle is likely to require some time to adapt to grow in tick tissues. In tick cell cultures, infection with known arboviruses does not cause any detectable CPE (Bell-Sakyi et al., 2012) and potential replication of any virus in tick cells must be investigated using other diagnostic tools such as real-time PCR and determination of endpoint titre in vertebrate cells. Previous attempts to propagate poxviruses in tick cells included a study with vaccinia virus in primary *Hyalomma dromedarii* cell cultures (Rehacek, 1965) and another study on vaccinia, ectromelia, fowl pox and orf viruses in the RA243 cell line (Munz et al., 1980). The time of incubation in the first study was eight days and in the second study five days. Neither of these studies resulted in poxvirus replication in tick cells detectable by endpoint titration in vertebrate cells. In the present study, based on experience obtained from propagation of the virus in mammalian cells, LSDV was incubated for a longer time with the tick cells, 35 days, which involved weekly changes of growth medium. This procedure resulted unavoidably in a loss of a few tick cells, possibly containing intracellular virus and a loss of extracellular virus in the growth medium. Therefore, in order to evaluate the effect of the medium changes on C_t_ values and virus titres, the following controls were included: C1 indicated the survival of the virus in L-15 and C2 in H-lac medium for 35 days. C1D and C2D demonstrated how much virus was lost due to the weekly changes of the growth medium irrespective of occurrence of viral replication. According to these results it was evident that the virus survived in embryo-derived RAE/CTVM1 and developing adult-derived RAN/CTVM3 and RA243 cells as well as it would have survived in growth medium without any tick cells and no replication of the virus occurred in *R. appendiculatus* cells.

Interestingly, survival of the virus in *R. (B.) decoloratus* cells propagated in L-15 (BDE/CTVM16 cells) and H-Lac (BDE/CTVM14 cells) medium decreased to below the detection limit of the virus titration assay, although CPE was still detected in one out of six wells in the 10^−1^ dilution row on the titration plate after 35 days in BDE/CTVM16 cells. The poor survival of the virus in *R. (B.) decoloratus* cells cannot be explained by the difference in the incubation temperature and the same virus isolate was used for all cell lines. Throughout the study, attempts to grow the virus in *R. (B.) decoloratus* cells resulted in higher final C_t_ values than for LSDV in *R. appendiculatus* cells. The only possible factor is the tick cells themselves and in this study we were not able to demonstrate with certainty why the virus did not survive as well in *R. (B.) decoloratus* cell cultures as in *R. appendiculatus* cells.

L-15 medium supported survival of the virus better than H-Lac medium. After incubation of the virus for 35 days the titre of the virus in L-15 had decreased by less than 1 log TCID_50_, whereas in H-Lac the titre had fallen by nearly 2.5 log TCID_50_. This may also have affected the survival of the virus in tick cell lines grown in the two different media. Moreover, if the virus was attached to the surface of the tick cells instead of being inside of the cells, some virus may have been lost when the cell debris was separated by centrifugation from the supernatant during the sample preparation for virus isolation, and the proportion of virus retained in the debris might differ between cell lines. The low cultivation temperature required by tick cells is likely to have a suppressing effect on the replication rate of a vertebrate virus (Rehacek, 1965). The tick cell lines used in this study were maintained at 28 °C, which is considerably lower than the average body temperature of cattle (38.5 °C) ideal for the replication of LSDV. During the first experiment in which the infected cells were incubated for the first seven days at 37 °C, *R. (B.) decoloratus* cells in particular started to deteriorate and it was not possible to incubate the infected tick cells longer at the higher temperature. When LSDV was kept in PBS (C3) at a similar temperature as the infected cells (28 °C), hardly any difference was detected in the C_t_ values determined on Days 0 and 35 pi whereas the virus titre decreased 2.29 log TCID_50_ over the 35 days of incubation; this illustrates, in addition to the exceptional stability of LSDV, how unreliable C_t_ values are as a measurement of virus viability and infectivity.

Tick cell lines are heterogeneous, comprising cells originating from different tissues of embryonic or developing adult ticks (Bell-Sakyi et al., 2012). In order to survive within moulting larvae or nymphs, the virus presumably has to establish infection in at least one cell type that does not undergo histolysis during the moulting process (Nuttall et al., 1994). It is likely that not all the cells present in the tick cell culture will support survival or growth of the virus which may have affected the results of the present study. In the cell lines originating from developing adults (RA243 and RAN/CTVM3), the digestive and excretory tissues were removed prior to initiation of the cell lines (Varma et al., 1975) which may have precluded growth of the virus as midgut cells are presumably the site where virus multiplication commences. On the other hand, the embryo-derived cell lines RAE/CTVM1, REE/CTVM28 and the two *R. (B.) decoloratus* cell lines may contain cells of midgut origin but equally did not support LSDV growth detectable in the present study. LSDV antigen was detected in haemocytes of intrastadially- and transstadially-infected *R. appendiculatus* (Lubinga et al., 2014a), indicating that the virus can infect these cell types which are present in all the tick cell lines tested (author's unpublished observation).

Most tick cell lines are known to permanently harbour endogenous tick viruses. Using transmission electron microscopy, endogenous reovirus-like particles were demonstrated in all the tick cell lines included in the present study except BDE/CTVM16, while putative nairovirus nucleic acid was detected by PCR in all the cell lines except REE/CTVM29. The *R. appendiculatus* cell line RA243 was PCR-positive for the endogenous orbivirus St Croix River virus, while all six cell lines were PCR-negative for flaviviruses (Alberdi et al., 2012). Very little is known about endogenous tick viruses which may affect the survival and replication of other viruses such as LSDV propagated in tick cell lines (Bell-Sakyi and Attoui, 2013).

In order to survive inside a tick, adaptation of the virus may not necessarily be required, provided the tick cells do not contain substances that are toxic to the virus. LSDV was able to survive for 35 days at 28 °C in PBS with a 48% decrease in the titre of the virus. However, similar C_t_ values detected on Days 0 and 35 pi clearly demonstrate that the real-time PCR method detects viral DNA from both virulent and non-infective viruses and C_t_ values cannot replace virus titration as a quantitative assay for viability of the virus.

Vertical transmission of LSDV by *R. (B.) decoloratus* ticks has been demonstrated (Tuppurainen et al., 2013a) and transstadial transmission by *A. hebraeum* adults, moulted from nymphs fed on experimentally-infected cattle, has been reported (Lubinga et al., 2013a). Survival of infectious LSDV in ticks was demonstrated when live virus was recovered after 90 days from moulting *A. hebraeum* nymphs following intracoelomic inoculation of virus and after 96 days from *R. (B.) decoloratus* larvae, originating from similarly infected fully engorged females (Lubinga et al., 2014b). Based on the evidence obtained from the present study, in addition to the previous investigations, we suggest that viable and infective LSDV may only survive in tick cells and tissues without actual replication; nevertheless this survival may be sufficient to result in transmission by the next life cycle stage following attachment and feeding on a susceptible host. Further immunohistochemical and ultrastructural studies are on-going to investigate whether the virus is able to enter inside tick cells *in vitro* or survives attached to the cell surface. The real-time PCR assay used in the present study targets the LSDV structural gene P32, thus quantifying virus DNA but not detecting nucleic acid associated with viral replication. At the time, a validated PCR quantifying a non-structural gene expressed during viral replication was not available. However, in future studies, improved molecular methods measuring viral mRNA associated with viral replication should be developed and utilised to determine if LSDV actually replicates in tick cells. In addition, more experiments are required in order to obtain statistical confirmation for the results of the present study.

In addition to infection by the oral route, it is possible that female ticks could obtain the virus during copulation. Venereal transmission of Crimean-Congo haemorrhagic fever virus has previously been reported in *Hyalomma truncatum* ticks (Gonzalez et al., 1992). During interrupted feeding on the skin of a viraemic host, the mouthparts of the males are likely to become contaminated with the virus. After finding a female ready for mating, the male tick places its capitulum next to her genital aperture and oscillates his mouthparts in the female genital opening several times to distend it prior to sperm transfer. The male grasps a spermatophore with his chelicerae and implants it in the female gonophore. The sack of sperm is then pushed into the female genital tract. Males copulate several times with the same or different females between repeated feeds (Varma, 1993). Therefore actual replication of the virus might not be required for transovarial transmission if the virus enters the female sex organs *via* male mouthparts and sperm.

Previous studies on transmission of LSDV by ticks have all utilised laboratory ticks fed on experimentally-infected cattle (Lubinga et al., 2014a,b,c; Lubinga et al., 2013a,b; Tuppurainen et al., 2013a,b; Tuppurainen et al., 2011). Here we report the presence of LSDV nucleic acid detected in naturally-infected ticks, collected from field cases of the disease in Egypt and South Africa. The high infection rates (33–100%) and, in some cases, high levels of viral DNA detected by qPCR in field ticks, combined with the experimental transmission studies, strongly suggest that ticks of several genera may be involved in the epidemiology of LSD. The results obtained from the field ticks indicate that potential vector capacity of *R. (B.) microplus* and *H. truncatum* in the transmission of LSDV should be investigated.

In summary, we were able to demonstrate that the virus survived *in vitro* in tick cell cultures for 35 days without losing its infectivity. No evidence was obtained for replication of LSDV in *R. appendiculatus*, *R. evertsi* or *R. (B.) decoloratus* cell lines. Presence of LSD viral DNA from *Rhipicephalus* and *Amblyomma* ticks, collected from naturally-infected animals, provides supporting evidence on the role of these tick genera in the transmission of LSDV. The results of this study indicate that intra- or extracellular survival of the virus in tick tissues is likely to be more important than actual replication of the virus in tick cells. However, the virus may be able to replicate under certain conditions that were not reproduced *in vitro* in this study such as during hot and humid seasons creating optimal conditions for both ticks and virus or in the presence of optimal growth or supplementary factors.

## Figures and Tables

**Table 1:** *Rhipicephalus* spp. tick cell lines tested for ability to support lumpy skin disease virus replication.

Tick cell line	Species and instar of origin	Medium	Reference
RAE/CTVM1	*R. appendiculatus* embryos	L-15	Bell-Sakyi (2004)
RA243	*R. appendiculatus* developing adults	L-15	Varma et al. (1975)
RAN/CTVM3	*R. appendiculatus* developing adults	H-Lac	Bekker et al. (2002)
REE/CTVM29	*R. evertsi* embryos	L-15	Alberdi et al. (2012)
BDE/CTVM16	*R. (B.) decoloratus* embryos	L-15	Bell-Sakyi (2004)
BDE/CTVM14	*R. (B.) decoloratus* embryos	H-Lac	Lallinger et al. (2010)

**Table 2:** Survival of lumpy skin disease virus in three *Rhipicephalus* spp. cell lines incubated at different temperatures in the first experiment. Cycle threshold (C_t_) values determined for samples collected on days 0 and 35 post-infection are presented for tick cells incubated with LSDV for 7 days at 37° and thereafter at 28 °C, or at 28 °C throughout.

Tick cell line	Incubation temperature	Day 0 (C_t_)	Day 35 (C_t_)
RAE/CTVM1	37°/28 °C	22.23	27.42
	28 °C throughout	21.59	27.02
REE/CTVM29	37°/28 °C	21.97	27.66
	28 °C throughout	25.94	27.84
BDE/CTVM16	37°/28 °C	21.03	35.14
	28 °C throughout	23.10	34.61
L-15 control	37°/28 °C	22.37	24.10
	28 °C throughout	22.78	24.11

**Table 3:** Virus titration and real-time polymerase chain reaction (PCR) results for five lumpy skin disease virus-infected *Rhipicephalus* spp. cell lines and cell-free controls incubated at 28 °C for 35 days in the second experiment.

	Medium	Cycle threshold (C_t_) values		Virus titre (TCID_50_)	
		0 dpi	35 dpi	0 dpi	35 dpi
*Tick cell line*					
RAE/CTVM1	L-15	23.69	30.59	5.63	3.0
RA243	L-15	23.43	30.80	5.63	3.5
RAN/CTVM3	H-Lac	24.31	30.13	5.63	2.5
BDE/CTVM16	L-15	25.46	34.09	5.63	a
BDE/CTVM14	H-Lac	25.52	30.43	5.63	b

*Cell-free control*					
C1 (undiluted)	L-15	23.34	24.65	5.63	4.84
C1D (diluted)	L-15	23.34	29.80	5.63	3.50
C2 (undiluted)	H-Lac	25.68	25.08	5.63	3.17
C2D (diluted)	H-Lac	24.27	34.04	5.63	2.34
C3 (undiluted)	PBS	24.34	24.03	5.63	3.34

a Below detectable levels, live virus isolated.

b Below detectable levels, no live virus isolated.

**Table 4:** Detection of lumpy skin disease viral DNA in field ticks collected from cattle during lumpy skin disease outbreaks in Egypt and South Africa. C_t_ values >39 were considered negative.

Sample location	Tick species and gender	Number of ticks (LSDV positive/total)	Mean C_t_ value of positive ticks
Menofilia, Egypt	*Rhipicephalus* (*Boophilus*) sp. female	4/4^a^	Not done
Pretoria, Gauteng, South Africa	*R. appendiculatus* female	9/9	35.84
	*R. appendiculatus* male	2/2	33.30
	*R. (B.) microplus* female	4/11	35.49
	*A. hebraeum* female	1/3	37.30
	*A. hebraeum* male	6/6	35.33
	*H. truncatum* female	4/4	33.01
Mnisi, Mpumalanga, South Africa	*Amblyomma* sp.^b^	2/6	33.67
	*Rhipicephalus (Boophilus)* sp. female	6/11	32.03

a Tested by gel-based PCR.

b Gender not recorded.
